# Body Mass Index and Gestational Weight Gain Are Associated with Maternal and Neonatal Outcomes Based on Chinese Women

**DOI:** 10.1155/2021/4542367

**Published:** 2021-11-24

**Authors:** Tingzhao Wang, Lichun Li, Chunchun Wu, Rong Cao, Qingli Li, Liji Yu, Youguo Chen

**Affiliations:** ^1^Department of Obstetrics and Gynecology, The First Affiliated Hospital of Soochow University, Suzhou 215006, China; ^2^Department of Obstetrics and Gynecology, Quanzhou First Hospital Affiliated to Fujian Medical University, Quanzhou 362000, China

## Abstract

The objective of the study is to analyze the association between early pregnancy body mass index (BMI), gestational weight gain (GWG), and maternal and neonatal outcomes. The retrospective cohort study was conducted at Quanzhou First Hospital Affiliated to Fujian Medical University from January 2018 to May 2021, with 552 women enrolled. Women were divided into the underweight group, normal weight group, overweight group, and obese group according to early pregnancy BMI. Univariate and multivariate logistic regression analyses were performed. The absolute risk of adverse maternal and neonatal outcomes in the early pregnancy BMI group was calculated to further analyze the association between GWG and adverse maternal and neonatal outcomes. Of the 552 women, 390 (70.65%) women had adverse maternal and neonatal outcomes. The result revealed that overweight was associated with increased risk of adverse maternal and neonatal outcomes (odds ratio (OR): 1.643, 95% confidence interval (CI): 1.006-2.684), maternal complications (OR: 1.937, 95% CI: 1.188-3.159), and large for gestational age (LGA) (OR: 1.905, 95% CI: 1.061-3.422). In the obese group, the risk of adverse maternal and neonatal outcomes (OR: 5.760, 95% CI: 1.997-16.786), maternal complications (OR: 3.112, 95% CI: 1.645-5.887), gestational diabetes mellitus (GDM) (OR: 2.943, 95% CI: 1.509-5.741), cesarean section (OR: 1.899, 95% CI: 1.002-3.599), and preterm delivery (OR: 4.752, 95% CI: 1.395-16.185) increased. Besides, there was an association between insufficient GWG and decreased risk of LGA (OR: 0.392, 95% CI: 0.187-0.826) and higher risk of preterm delivery (OR: 2.818, 95% CI: 1.171-6.784). This study demonstrates that BMI and GWG are related to maternal and neonatal outcomes. It is necessary to regularly monitor the weight of pregnant women during pregnancy. And regional guidelines for GWG also need to be explored.

## 1. Introduction

In recent years, the prevalence of overweight and obesity among women at reproductive age is increasing [1, 2]. The 2011 Pregnancy Nutrition Surveillance on maternal health indicators showed a prevalence of 4.5% and 53.7% of women having a prepregnancy body mass index (BMI) in the underweight and overweight category, respectively [3]. In China, the national nutrition survey revealed that being overweight and obese for women aged 18-44 reached 21.8% and 6.1%, respectively, and that there was an increasing trend particularly in women of childbearing age [4]. Being overweight or obese increases the risk of diabetes, high blood pressure, and disorders of fetal growth and is even linked to the development of many cancers, which is a serious global public health challenge [5]. The repercussion of this rising increase in weight on maternal and neonatal outcomes is imperative to be studied.

Previous studies have revealed that maternal outcomes are related to obesity in pregnant women, including gestational diabetes mellitus (GDM), pregnancy-induced hypertension, preeclampsia, and postpartum hemorrhage [6–8]. Nevertheless, studies examining the effect of pregnant woman's BMI on the neonatal and parturition outcome are scanty. The relationship between BMI and maternal and neonatal outcomes has not been fully studied. Pregnancy weight status may affect the total gestational weight gain (GWG), which is defined as weight just before delivery minus weight just before conception [9]. GWG reflects a variety of characteristics, including the accumulation of maternal fat, fluid swelling, and the growth of the fetus, placenta, and uterus [10]. GWG is necessary to ensure fetal health, but a study found that excessive GWG was associated with adverse outcomes [11]. Currently, most of the evidence on GWG values comes from Western or high-income countries [12, 13]. The maternal and perinatal outcomes of GWG in developing countries still need to be elucidated. It is of particular relevance to study the effects of BMI and GWG on pregnancy and the newborn and to develop a reasonable pregnancy weight control plan.

The principal purpose of this study was to examine the effect of two anthropometric indicators (BMI and GWG) on maternal and neonatal outcomes in a sample of Chinese women. Besides, we calculated the absolute risk of adverse maternal and infant outcomes for GWG in the early pregnancy BMI to further analyze the association of GWG with maternal and neonatal adverse outcomes. This study may provide a reference for regular weight monitoring during pregnancy, appropriate weight gain during pregnancy, and solutions to reduce adverse maternal and neonatal outcomes.

## 2. Methods

### 2.1. Study Design and Participants

This study was a retrospective cohort study. We retrospectively collected data from 552 singleton pregnancies in the Quanzhou First Hospital Affiliated to Fujian Medical University from January 2018 to May 2021. The inclusion criteria were as follows: (1) women with singleton pregnancy and (2) regular postnatal examinations. Exclusion criteria were as follows: (1) women with chronic hypertension or pregestational diabetes; (2) women with previous pregnancy complications; (3) height < 140 cm; (4) early pregnancy weight < 35 kg; (5) gestation < 22 weeks or >44 weeks; and (6) fetal malformations, fetal reduction, or fetal chromosomal abnormalities.

### 2.2. Data Collection

Data in this study were obtained from computer tracking systems or medical records, including (1) sociodemographic data: age (year) and education level; (2) adverse maternal outcomes: maternal complications including GDM, gestational hypertension, proteinuria, thrombocytopenia, impaired liver function, preeclampsia, placenta previa, chorioamnionitis, and parturition outcome (cesarean delivery); and (3) adverse neonatal outcomes: premature delivery, large for gestational age (LGA), small for gestational age (SGA), more than 48 h in the neonatal intensive care unit (NICU), Apgar score < 8, and other neonatal complications.

### 2.3. BMI and GWG

All participants were weighed at 12 ± 1 weeks of gestation and were categorized into four subgroups by the range of BMI according to the WHO classifications: underweight (BMI < 18.5 kg/m^2^), normal (BMI 18.5-25 kg/m^2^), overweight (BMI 25-30 kg/m^2^), and obese (BMI ≥ 30 kg/m^2^) [14].

GWG was defined as the difference between the final body weight before delivery and the prepregnancy body weight of mothers. GWG was further categorized into three subgroups according to the Institute of Medicine (IOM) [15] guidelines of recommended weight gain during pregnancy as inadequate, within the range, or excessive compared to the recommendations for different BMI categories (underweight: 12.5-18 kg; normal weight: 11.5-16 kg; overweight: 7-11.5 kg; and obese: 5-9 kg) [14].

### 2.4. Adverse Maternal and Neonatal Outcomes

The main outcome of the analysis is the composite of any adverse outcome, which was defined as the presence of at least one of the following outcomes: GDM, gestational hypertension, preeclampsia, eclampsia, placenta previa, chorioamnionitis, cesarean delivery, premature delivery, LGA, SGA, more than 48 h in NICU, Apgar score < 8, and other neonatal complications. The definition of preterm birth is less than 37 weeks of gestational age at birth. Use the Nordic reference chart to calculate gender-adjusted birth weight and age-adjusted SD score during pregnancy. SGA at birth was defined as gender- and gestational age-adjusted birth weight less than 10th and greater than 90th percentile [11]. LGA was referred to a weight above the 90th percentile for gestational age [16].

### 2.5. Statistical Analysis

The measurement data of normal distribution were described as mean ± standard deviation (mean ± SD), and analysis of variance (ANOVA) was used for comparison between groups. Nonnormal distribution was exhibited as *M* (*Q*_1_, *Q*_3_), and the Kruskal-Wallis *H* rank sum test was used for comparison between groups. Enumeration data were described in terms of the number of cases and composition ratio (*N* (%)). The chi-square test or Fisher's exact probability method was used for comparison between groups. The risk estimation was reported as an odds ratio (OR) with 95% confidence interval (95% CI). Variables that had statistical significance in univariate analysis and had influence on maternal and neonatal adverse outcomes obtained from literature were included in multivariate logistic regression analysis to explore the influence of BMI in early pregnancy and GWG on maternal and neonatal adverse outcomes. The absolute risk of adverse maternal and neonatal outcomes in the early pregnancy BMI group was calculated to further analyze the association between GWG and adverse maternal and neonatal outcomes. All statistical tests were conducted by the two-tailed test, and *P* < 0.05 was considered statistically significant. All statistical analyses were conducted using SAS Statistical Software version 9.4 (SAS Institute, Cary, NC, USA).

## 3. Results

### 3.1. Basic Characteristics of Included Pregnant Women

A total of 552 singleton women were included in this study, with an average age of 29.70 ± 4.42 years. In the early weeks of pregnancy, 120 (21.74%) women were underweight, 209 (37.86%) women were normal weight, 164 (29.71%) women were overweight, and 59 (10.69%) women were obese. During pregnancy, 140 women (25.36%) had insufficient GWG, 253 women (45.83%) had suitable GWG, and 159 women (28.80%) had excessive GWG. Fourteen women (2.54%) had a history of premature birth, 205 (37.14%) had a history of miscarriage, and 27 (4.89%) had a history of medication. Adverse maternal and neonatal outcomes occurred in 390 (70.65%) women, 277 (50.18%) had cesarean delivery, and 328 (64.86%) had neonatal adverse outcomes. Basic characteristics of the included population are shown in Table 1.

### 3.2. Comparison of Related Characteristics of Maternal BMI Groups in Early Pregnancy

The analysis results showed that the age (*F* = 15.377, *P* < 0.001), the number of pregnancies (*χ*^2^ = 20.350, *P* < 0.001), the abortion history (*χ*^2^ = 19.032, *P* < 0.001), the medication history (*χ*^2^ = 11.295, *P* = 0.010), the maternal complications (*χ*^2^ = 22.224, *P* < 0.001), the cesarean section (*χ*^2^ = 28.799, *P* < 0.001), the adverse neonatal outcomes (*χ*^2^ = 32.349, *P* < 0.001), and the adverse maternal and neonatal outcomes (*χ*^2^ = 36.274, *P* < 0.001) of the four groups were different, and the difference is statistically significant (Table 2).

### 3.3. Effects of BMI in Early Pregnancy on Maternal and Neonatal Adverse Outcomes

The result showed that underweight women had a reduced risk of adverse maternal and neonatal outcomes (OR: 0.616, 95% CI: 0.388-0.975) and a reduced risk of cesarean section delivery (OR: 0.525, 95% CI: 0.329-0.839) compared with normal weight women. Compared with women in the normal weight group, overweight women had an increased risk of adverse maternal and neonatal outcomes (OR: 1.926, 95% CI: 1.198-3.094), maternal complications (OR: 2.140, 95% CI: 1.329-3.446), cesarean section (OR: 1.618, 95% CI: 1.070-2.448), and LGA (OR: 2.230, 95% CI: 1.271-3.912). Similarly, obesity women had an increased risk of adverse maternal and neonatal outcomes (OR: 6.924, 95% CI: 2.411-19.883), maternal complications (OR: 3.678, 95% CI: 1.980-6.832), GDM (OR: 3.534, 95% CI: 1.855-6.733), cesarean section (OR: 2.294, 95% CI: 1.247-4.222), LGA (OR: 2.293, 95% CI: 1.095-4.802), and preterm birth (OR: 3.268, 95% CI: 1.054-10.132).

After adjusting for age, history of miscarriage, number of pregnancies, and medication history, overweight women had an increased risk of adverse maternal and neonatal outcomes (OR: 1.643, 95% CI: 1.006-2.684), an increased risk of maternal complications (OR: 1.937, 95% CI: 1.188-3.159), and an increased risk of having LGA infants (OR: 1.905, 95% CI: 1.061-3.422). In the obese group, the risk of adverse maternal and neonatal outcomes (OR: 5.760, 95% CI: 1.997-16.786), maternal complications (OR: 3.112, 95% CI: 1.645-5.887), GDM (OR: 2.943, 95% CI: 1.509-5.741), cesarean section (OR: 1.899, 95% CI: 1.002-3.599), and preterm delivery (OR: 4.752, 95% CI: 1.395-16.185) increased (Table 3).

### 3.4. Effects of GWG on Maternal and Neonatal Adverse Outcomes

The analysis results indicated that compared with women with appropriate GWG, women with insufficient GWG had a decreased risk of having LGA infants (OR: 0.426, 95% CI: 0.205-0.884) and women with excessive GWG during pregnancy had an increased risk of cesarean delivery (OR: 1.522, 95% CI: 1.020-2.270). After adjusting for the variables age, education level, and history of miscarriage, insufficient GWG was associated with the decreased risk of having LGA infants (OR: 0.392, 95% CI: 0.187-0.826) and increased risk of preterm birth (OR: 2.818, 95% CI: 1.171-6.784) (Table 4).

### 3.5. Absolute Risk of Adverse Outcomes with GWG in the Early Pregnancy BMI Group

The result showed that among women categorized as underweight, the absolute risk of adverse maternal and neonatal outcomes increased from 48.57% for suitable GWG to 100% for excessive GWG and the absolute risk was highest for cesarean delivery (highest risk: 85.71% for excessive GWG). The absolute risk of adverse maternal and neonatal outcomes increased from 65.71% with insufficient GWG to 73.40% with appropriate GWG among women categorized as normal weight, and the absolute risk of preterm birth is lowest (lowest risk: 85.71% for suitable GWG). Among women categorized as overweight, the absolute risk of adverse maternal and neonatal outcomes increased from 72.73% with appropriate GWG to 83.54% with excessive GWG and the absolute risk of cesarean section was the highest (highest risk: 63.29% for excessive GWG). And the absolute risk of adverse maternal and neonatal outcomes decreased from 100% of underweight gain to 95.65% of overweight gain among obese women.  Table 5 depicts the absolute risk of adverse outcomes with GWG in the early pregnancy BMI group.

## 4. Discussion

Contradictory results between early pregnancy BMI, GWG, and maternal and neonatal outcomes have occurred in many cases, and most studies were conducted in high-income countries. We examined the association between BMI, GWG, and maternal and neonatal outcomes based on Chinese populations. Our findings displayed that high maternal BMI was associated with increases in risk of pregnancy complications, LGA, GDM, cesarean section, and premature delivery. Besides, compared with women who gained suitable GWG during pregnancy, women who did not gain enough weight during pregnancy had a decreased risk of having LGA infants and had an increased risk of preterm birth. In addition, results from absolute risk indicated that women with suitable GWG in the normal weight group had a high risk of adverse maternal and neonatal outcomes.

In our findings, obesity and overweight increased the risk of adverse maternal and neonatal outcomes, including LGA, GDM, cesarean section, and premature delivery. A population-based cohort study of almost 1.6 million singleton deliveries in Sweden from 1992 through 2010 showed an increased risk for preterm infants in overweight and obese pregnant women [17]. In this study, overweight women had an increased risk of pregnancy complications and obese women had an increased risk of GDM. Yong et al. [18] revealed that overweight/obese was independently associated with the risk of GDM. We observed a growing association between BMI and LGA outcomes, which was supported by other studies [4, 19]. Nowak et al. [19] demonstrated that underweight women were less likely to have a SGA newborn while obese mothers had a higher risk of a LGA newborn. Obesity is characterized by inflammatory upregulation, which is associated with proinflammatory cytokines and adipokines and alterations of the hypothalamic-pituitary-adrenal axis, being responsible for releasing corticotrophin-releasing hormone [20, 21]. In high values, it is known as a risk factor for premature rupture of membranes, preterm delivery, eclampsia, and pregnancy-induced hypertension [17]. This may be the underlying mechanism that explains adverse maternal and child outcome risk in women with higher BMI.

Compared with suitable GWG, insufficient GWG was associated with higher risk for preterm birth in our findings. Similarly, the result from a study [22] indicated that low GWG was associated with an increased risk of all subtypes of preterm birth compared with normal GWG, especially in early spontaneous preterm births, where the risk was doubled. Goldstein et al. noted that low GWG was a risk factor for preterm birth [23]. It is hypothesized that low GWG contributes to preterm birth through deficiencies in micro- and macronutrients, which increase the risk of preterm birth [24]. Nonetheless, Silva et al. [25] demonstrated that the rate of GWG was associated with preterm birth risk depending on the initial BMI. Prepregnancy BMI might play an important role in the relationship between GWG and preterm birth. The single and combined effects of BMI and GWG on the risk of adverse maternal and neonatal outcomes should also be considered. In general, clinicians use the IOM guidelines to educate pregnant women about the best GWG recommendations for different BMI categories [26]. However, we found that normal weight women with suitable GWG had a high risk of adverse maternal and neonatal outcomes, suggesting that the use of GWG guidelines may need to be reconsidered for individual difference. Further study may also be needed to determine applicable guidelines for Chinese women.

The data were rigorously obtained from the computer tracking system or medical records, ensuring the accuracy of the study data. However, several limitations should be taken into consideration. Our study was limited by its observational, retrospective, and single-center design, and the findings may not be generalizable to all pregnant women. Future studies should enroll participants from other hospitals in order to take into consideration regional, educational, and social differences.

## 5. Conclusions

This study demonstrated that BMI and GWG were associated with adverse maternal and neonatal outcomes. In clinical practice, pregnant women should be instructed to have a clear understanding of weight gain, have regular examination during pregnancy, and reasonably control weight, reducing the risk of pregnancy complications and neonatal adverse outcomes.

## Figures and Tables

**Table 1:** Basic characteristics of the included population.

Variables	(n = 552)
Age (year), mean ± SD	29.70 ± 4.42
Early pregnancy BMI groups (kg/m^2^), *n* (%)	
Underweight (BMI < 18.5)	120 (21.74)
Normal weight (18.5 ≤ BMI < 23)	209 (37.86)
Overweight (23 ≤ BMI < 27.5)	164 (29.71)
Obesity (BMI ≥ 27.5)	59 (10.69)
GWG (kg), *n* (%)	
Insufficient	140 (25.36)
Suitable	253 (45.83)
Excessive	159 (28.80)
Educational level, *n* (%)	
Junior high school and below	120 (21.74)
High school or vocational high school	155 (28.08)
Graduate degree and above	277 (50.18)
Number of pregnancies, *M* (Q_1_, Q_3_)	2 (1, 3)
History of premature birth, *n* (%)	14 (2.54)
History of miscarriage, *n* (%)	205 (37.14)
History of medication, *n* (%)	27 (4.89)
Maternal complications, *n* (%)	147 (26.63)
GDM	105 (19.02)
Pregnancy-induced hypertension	6 (1.09)
Proteinuria	1 (0.18)
Thrombocytopenia	6 (1.09)
Impaired liver function	2 (0.36)
Preeclampsia	39 (7.07)
Placenta previa	2 (0.36)
Chorioamnionitis	5 (0.91)
Parturition outcome (cesarean delivery), *n* (%)	277 (50.18)
Adverse neonatal outcome, *n* (%)	358 (64.86)
Premature delivery	28 (5.07)
LGA	84 (15.22)
SGA	39 (7.07)
In NICU more than 48 h	21 (3.80)
Apgar score < 8	
1 min	13 (2.36)
5 min	3 (0.45)
10 min	1 (0.18)
Other neonatal complications	63 (11.41)

Notes: BMI: body mass index; GWG: gestational weight gain; GDM: gestational diabetes mellitus; LGA: large for gestational age; SGA: small for gestational age; NICU: neonatal intensive care unit.

**Table 2:** Comparison of related characteristics of maternal BMI groups.

Variables	Total	Groups				Statistics	*P*
		Underweight (*n* = 120)	Normal weight (*n* = 209)	Overweight (*n* = 164)	Obesity (*n* = 59)		
Age (year), mean ± SD	29.70 ± 4.42	28.03 ± 3.88	29.18 ± 4.13	31.01 ± 4.72	31.36 ± 4.08	*F* = 15.377	<0.001
Educational level, *n* (%)						*χ* ^2^ = 9.009	0.173
Junior high school and below	120 (21.74)	18 (15.00)	41 (19.62)	47 (28.66)	14 (23.73)		
High school or vocational high school	155 (28.08)	37 (30.83)	57 (27.27)	44 (26.83)	17 (28.81)		
Graduate degree and above	277 (50.18)	65 (54.17)	111 (53.11)	73 (44.51)	28 (47.46)		
Number of pregnancies, *M* (*Q*_1_, *Q*_3_)	2 (1, 3)	1 (1, 2)	2 (1, 3)	2 (1, 3)	2 (2, 3)	*χ* ^2^ = 20.350	<0.001
History of premature birth, *n* (%)	14 (2.54)	3 (2.50)	5 (2.39)	4 (2.44)	2 (3.39)	*χ* ^2^ = 0.198	0.978
History of miscarriage, *n* (%)	205 (37.14)	27 (22.50)	76 (36.36)	72 (43.90)	30 (50.85)	*χ* ^2^ = 19.032	<0.001
History of medication, *n* (%)	27 (4.89)	3 (2.50)	9 (4.31)	7 (4.27)	8 (13.56)	*χ* ^2^ = 11.295	0.010
Adverse maternal and neonatal outcomes							
Maternal complication, *n* (%)	147 (26.63)	27 (22.50)	39 (18.66)	54 (32.93)	27 (45.76)	*χ* ^2^ = 22.224	<0.001
Parturition outcome (cesarean delivery), *n* (%)	277 (50.18)	39 (32.50)	100 (47.85)	98 (59.76)	40 (67.80)	*χ* ^2^ = 28.799	<0.001
Adverse neonatal outcome, *n* (%)	358 (64.86)	58 (48.33)	128 (61.24)	122 (74.39)	50 (84.75)	*χ* ^2^ = 32.349	<0.001
Adverse maternal and neonatal outcomes, *n* (%)	390 (70.65)	66 (55.00)	139 (66.51)	130 (79.27)	55 (93.22)	*χ* ^2^ = 36.274	<0.001

**Table 3:** BMI in early pregnancy on maternal and neonatal adverse outcomes.

Outcomes	Early pregnancy BMI	Univariate analysis		Multivariate analysis	
		OR (95% CI)	*P*	OR (95% CI)	*P*
Adverse maternal and neonatal outcomes					
Total					
	Underweight	0.616 (0.388-0.975)	0.039	0.691 (0.429-1.112)	0.128
	Normal weight	Ref		Ref	
	Overweight	1.926 (1.198-3.094)	0.007	1.643 (1.006-2.684)	0.048
	Obesity	6.924 (2.411-19.883)	<0.001	5.760 (1.977-16.786)	0.001
Maternal complications					
	Underweight	1.266 (0.729-2.198)	0.403	1.395 (0.795-2.446)	0.246
	Normal weight	Ref		Ref	
	Overweight	2.140 (1.329-3.446)	0.002	1.937 (1.188-3.159)	0.008
	Obesity	3.678 (1.980-6.832)	<0.001	3.112 (1.645-5.887)	0.001
GDM					
	Underweight	0.615 (0.304-1.244)	0.176	0.671 (0.328-1.372)	0.274
	Normal weight	Ref		Ref	
	Overweight	1.668 (0.989-2.814)	0.055	1.467 (0.855-2.516)	0.164
	Obesity	3.534 (1.855-6.733)	<0.001	2.943 (1.509-5.741)	0.002
Parturition outcome					
Cesarean delivery					
	Underweight	0.525 (0.329-0.839)	0.007	0.603 (0.370-0.981)	0.042
	Normal weight	Ref		Ref	
	Overweight	1.618 (1.070-2.448)	0.023	1.312 (0.848-2.028)	0.183
	Obesity	2.294 (1.247-4.222)	0.008	1.899 (1.002-3.599)	0.049
Adverse neonatal outcome					
	Underweight	0.843 (0.511-1.391)	0.503	0.883 (0.528-1.475)	0.634
	Normal weight	Ref		Ref	
	Overweight	1.522 (0.989-2.340)	0.056	1.433 (0.917-2.240)	0.115
	Obesity	1.481 (0.812-2.700)	0.201	1.258 (0.671-2.359)	0.474
LGA					
	Underweight	0.581 (0.251-1.343)	0.204	0.673 (0.287-1.579)	0.363
	Normal weight	Ref		Ref	
	Overweight	2.230 (1.271-3.912)	0.005	1.905 (1.061-3.422)	0.031
	Obesity	2.293 (1.095-4.802)	0.028	1.709 (0.784-3.726)	0.178
Premature delivery					
	Underweight	1.519 (0.498-4.629)	0.462	1.494 (0.477-4.676)	0.491
	Normal weight	Ref		Ref	
	Overweight	1.676 (0.610-4.599)	0.316	2.512 (0.838-7.529)	0.100
	Obesity	3.268 (1.054-10.132)	0.040	4.752 (1.395-16.185)	0.013
Other neonatal complications					
	Underweight	0.625 (0.280-1.393)	0.250	0.570 (0.253-1.281)	0.173
	Normal weight	Ref		Ref	
	Overweight	1.257 (0.682-2.320)	0.464	1.418 (0.756-2.659)	0.277
	Obesity	1.038 (0.423-2.543)	0.937	1.218 (0.484-3.062)	0.675

Notes: multivariate analysis was adjusted for age, education level, history of miscarriage, number of pregnancies, and medication history. BMI: body mass index; GDM: gestational diabetes mellitus; LGA: large for gestational age; OR: odds ratio.

**Table 4:** Effects of GWG on maternal and neonatal adverse outcomes.

Outcomes	GWG	Univariate analysis		Multivariate analysis	
		OR (95% CI)	*P*	OR (95% CI)	*P*
Adverse maternal and neonatal outcomes					
Total					
	Insufficient	0.976 (0.627-1.520)	0.915	0.899 (0.567-1.425)	0.651
	Appropriate	Ref		Ref	
	Excessive	1.525 (0.969-2.399)	0.068	1.497 (0.937-2.393)	0.091
Maternal complications					
	Insufficient	1.083 (0.686-1.709)	0.733	1.039 (0.654-1.651)	0.872
	Appropriate	Ref		Ref	
	Excessive	0.765 (0.482-1.215)	0.257	0.737 (0.461-1.179)	0.203
GDM					
	Insufficient	1.444 (0.875-2.383)	0.151	1.393 (0.836-2.320)	0.203
	Appropriate	Ref		Ref	
	Excessive	0.840 (0.493-1.431)	0.521	0.822 (0.478-1.413)	0.479
Parturition outcome					
Cesarean delivery					
	Insufficient	0.961 (0.635-1.453)	0.849	0.886 (0.573-1.371)	0.588
	Appropriate	Ref		Ref	
	Excessive	1.522 (1.020-2.270)	0.040	1.492 (0.979-2.274)	0.062
Adverse neonatal outcome					
	Insufficient	0.876 (0.562-1.366)	0.559	0.839 (0.534-1.318)	0.447
	Appropriate	Ref		Ref	
	Excessive	1.045 (0.688-1.588)	0.836	1.060 (0.692-1.622)	0.790
LGA					
	Insufficient	0.426 (0.205-0.884)	0.022	0.392 (0.187-0.826)	0.014
	Appropriate	Ref		Ref	
	Excessive	1.412 (0.847-2.354)	0.186	1.424 (0.840-2.413)	0.189
Premature delivery					
	Insufficient	2.252 (0.981-5.171)	0.056	2.818 (1.171-6.784)	0.021
	Appropriate	Ref		Ref	
	Excessive	0.568 (0.178-5.171)	0.340	0.427 (0.128-1.422)	0.166
Other neonatal complications					
	Insufficient	0.796 (0.08-1.552)	0.503	0.805 (0.411-1.577)	0.527
	Appropriate	Ref		Ref	
	Excessive	0.914 (0.493-1.696)	0.776	0.933 (0.501-1.739)	0.828

Notes: multivariate analysis was adjusted for age, education level, and history of miscarriage. GWG: gestational weight gain; GDM: gestational diabetes mellitus; LGA: large for gestational age; OR: odds ratio.

**Table 5:** Absolute risk of adverse maternal and neonatal outcomes.

Variables	Underweight (*n* = 120)			Normal weight (*n* = 209)			Overweight (*n* = 164)			Obesity (*n* = 59)		
	Insufficient (*n* = 43)	Appropriate (*n* = 70)	Excessive (*n* = 7)	Insufficient (*n* = 70)	Appropriate (*n* = 94)	Excessive (*n* = 45)	Insufficient (*n* = 19)	Appropriate (*n* = 66)	Excessive (*n* = 79)	Insufficient (*n* = 8)	Appropriate (*n* = 23)	Excessive (*n* = 28)
Adverse maternal and neonatal outcomes	25 (58.14)	34 (48.57)	7 (100.00)	46 (65.71)	69 (73.40)	24 (53.33)	16 (84.21)	48 (72.73)	66 (83.54)	8 (100.00)	22 (95.65)	25 (89.29)
GDM	5 (11.63)	6 (8.57)	1 (14.29)	18 (25.71)	11 (11.70)	3 (6.67)	7 (36.84)	17 (25.76)	14 (17.72)	4 (50.00)	12 (52.17)	7 (25.00)
Cesarean delivery	18 (41.86)	15 (21.43)	6 (85.71)	32 (45.71)	50 (53.19)	18 (40.00)	10 (52.63)	38 (57.58)	50 (63.29)	5 (62.50)	17 (73.91)	18 (64.29)
LGA	0 (0.00)	5 (7.17)	3 (42.86)	6 (8.57)	12 (12.77)	6 (13.33)	3 (15.79)	16 (24.24)	19 (24.05)	1 (12.50)	7 (30.43)	6 (21.43)
Premature delivery	4 (9.30)	2 (2.86)	0 (0.00)	5 (7.14)	1 (1.06)	1 (2.22)	2 (10.53)	5 (7.58)	2 (2.53)	2 (25.00)	3 (13.04)	1 (3.57)
Other complications	4 (9.30)	4 (5.71)	1 (14.29)	5 (7.14)	14 (14.89)	5 (11.11)	5 (26.32)	9 (13.64)	9 (11.39)	0 (0.00)	4 (17.39)	3 (10.71)

Notes: GDM: gestational diabetes mellitus; LGA: large for gestational age.

## Data Availability

No data are available for this manuscript.
